# Retracted: Effect of Supplementation of Vitamin D in Patients with Periodontitis Evaluated before and after Nonsurgical Therapy

**DOI:** 10.1155/2023/9850874

**Published:** 2023-09-27

**Authors:** BioMed Research International

This article has been retracted by Hindawi following an investigation undertaken by the publisher. This investigation has uncovered evidence that the peer review process was compromised. We cannot, therefore, vouch for its reliability. Please note that this notice is intended solely to alert readers that the peer review of this article is compromised.

We have subsequently provided the authors with the opportunity to re- submit their article and undergo a new and independent peer-review process.

Wiley and Hindawi regret that the usual quality checks did not identify these issues before publication and have since put additional measures in place to safeguard research integrity.

We wish to credit our Research Integrity and Research Publishing teams and anonymous and named external researchers and research integrity experts for contributing to this investigation.

The corresponding author, as the representative of all authors, has been given the opportunity to register their agreement or disagreement to this retraction. We have kept a record of any response received.
